# Cardiac Surgery for Patients Admitted to the Cardiac Intensive Care Unit: Worth the Risk∗

**DOI:** 10.1016/j.jacadv.2023.100280

**Published:** 2023-03-31

**Authors:** Ryan A. Watson, Russell D. Rosenberg

**Affiliations:** a Division of Cardiolovascular Medicine at Allegheny Health Network and Drexel College of Medicine, Pittsburgh, Pennsylvania, USA; b Division of Cardiovascular Medicine, Penn Presbyterian Medical Center, University of Pennsylvania Perelman School of Medicine, Philadelphia, Pennsylvania, USA

**Keywords:** cardiac intensive care unit, cardiac surgery, mechanical circulatory support, mortality

**Figure:**
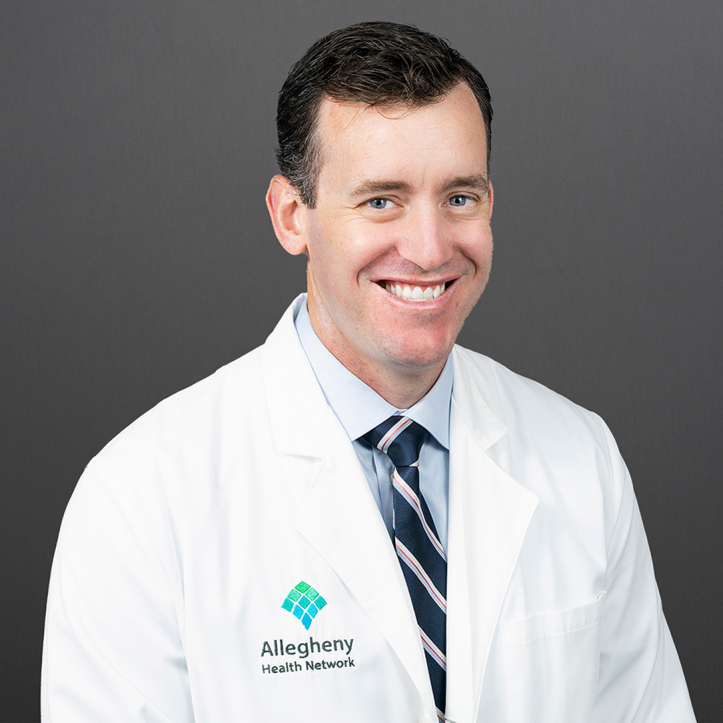

The cardiac intensive care unit (CICU) was developed in the 1960s primarily to admit patients with unstable arrhythmias in the setting of an acute myocardial infarction. In today’s CICU, patients frequently have more noncardiac comorbidities, making their clinical presentation more complex and, thus, their care more nuanced.^1^ Increasing patient complexity and a higher proportion of noncardiac illnesses prompted a movement to train cardiologists with specialized critical care skills to better care for this patient population. With the advancement of therapeutic interventions including mechanical circulatory support (MCS), a multidisciplinary team is paramount to improve patient outcomes.

The heart team model was created to bring together different specialties, each with their own expertise, to provide comprehensive care and facilitate individualized treatment. This model has been successfully implemented in various conditions including shock, pulmonary embolism, complex coronary disease, and aortic syndromes to improve patient outcomes.^2^ In the medically complex CICU patient, the heart team plays an integral role in the initial management, stabilization, and preoperative evaluation. With a paucity of data in this patient population, we are forced to rely on local expertise within the heart team to guide management strategies and therapeutic intervention. Without high-quality data, the prevailing and reasonable thought may be that medically ill and complex patients may not survive cardiac surgical interventions.

In this issue of *JACC: Advances*, Metkus et al^3^ analyzed 10,321 patients admitted to the CICU from 2017 to 2020 at 29 hospitals using the Critical Care Cardiology Trial Network (CCCTN) database to determine the in-hospital mortality of patients who underwent cardiac surgery directly from the CICU. The CCCTN is a prospective research network of tertiary care CICUs within North America established to provide an improved understanding of their unique patient population.^4^ The investigators used this database to describe the patient population and in-hospital mortality of critically ill patients who required cardiac surgical interventions directly from their stay in the CICU.

This observational study provides important insight into the composition of patients admitted to tertiary care CICUs across North America. These patients had significant comorbidities. For example, >80% of patients had a left ventricular ejection fraction ≤40%, and 20% of patients were on hemodialysis. They were acutely ill with over one-third presenting with shock, more than half requiring vasoactive therapy, approximately one-third requiring MCS, and over one-third requiring mechanical ventilation. Despite their critical illness, approximately 1 in 12 patients (8.6% of all admissions to CICUs) underwent cardiothoracic surgery during the 4-year study period. The majority of surgical interventions performed were coronary artery bypass graft (46%) followed by transplant or ventricular assist device (VAD) (23%).

The overall mortality rate of patients undergoing surgical intervention was lower than that of patients who were treated medically (9.1% vs 11.9%). In another large national database study, CICU mortality has been described to be lower than what was seen with the CCCTN data.^5^ This may very well be due to the referral bias at tertiary care centers who frequently accept critically ill patients requiring a higher level of care who remain at high risk of decompensation. The lower mortality seen in the surgical arm may represent the heart team’s ability to appropriately select surgical patients at reasonable perioperative risk, and for that, the heart team should be commended. Despite an improved mortality as compared to the nonsurgical arm, the surgical mortality rates described here in the CICU population in this paper remain higher than those of patients who present for cardiac surgical intervention and do not require initial ICU care.^6^ With highly acute and critically ill patients, risk factor modification and patient selection will remain paramount to improving surgical outcomes in this population. In-hospital mortality was highest in those patients requiring acute valvular surgery (12.3%) followed by patients undergoing VAD placement/transplant (11%). In a logistical regression analysis, patients who required MCS and/or renal replacement therapy had the highest risk of in-hospital mortality.

The rates of MCS were significantly higher in patients undergoing surgical intervention (35.9%) than in noncardiac surgery patients (9.8%). The indication for MCS was most commonly shock, yet for those patients referred for coronary artery bypass graft, MCS was more commonly placed for critical left main or severe coronary artery disease. The use of percutaneous MCS has gained considerable interest in recent years with the advent of new technologies to better support the failing ventricle. While the overall usage of MCS appears similar over the last decade, there has been a decrease in the usage of intra-aortic balloon pump in favor of more supportive devices such as Impella (Abiomed), Tandem Hearts (Cardiac Assist Inc), and percutaneous venoarterial extracorporeal membrane oxygenation.^7^ Even with these newer devices, mortality remains high with a lack of strong data supporting their use in all-comers. Despite adjusting for illness severity, MCS was associated with higher mortality in this study. The risks and benefits of using MCS must be assessed on an individualized patient level, as the sickest patients are the most likely to receive temporary mechanical support but are also the most likely to have poor outcomes or complications.

The high MCS usage (65.2% of all VAD/transplant patients) must also be considered in the context of the high VAD/transplant rate in this surgical population. In 2018, the United Network for Organ Sharing changed its donor heart allocation system to give preference to patients requiring MCS for transplantation. While the goal was to improve outcomes and equity among patients on the transplant list, there has been an overall increase in the usage of MCS at transplant centers. This has led to an obligate increase in CICU admissions to manage the MCS prior to surgical intervention.^8^ Due to the high rate of VAD/transplant in the patient population described, the risk related to MCS is not generalizable to nontransplant institutions where that confounding incentive may not exist.

The study is not without limitations. First, the CCCTN database is observational, and thus, causality cannot be determined. Nonetheless, this article gives further insight into the patient population in tertiary care CICUs and their relative risk regarding surgical outcomes. While these data cannot be generalized to all hospitals, it bolsters the importance of a strong referral base to tertiary care CICUs where specialized, multidisciplinary care can be achieved and outcomes can be improved.

Next, only patients who underwent surgery directly from the CICU were included in this study. Patients who were stabilized and transferred from the CICU and later underwent surgical intervention were not included. A comparison of patients requiring a more urgent cardiac surgery during their initial presentation and those able to be downgraded out of the CICU before surgery would be beneficial to the medical literature and our understanding around preoperative medical care. It would seem intuitive that if critically ill patients could be medically optimized to the point of no longer requiring CICU-level care prior to surgery, their outcomes would improve.

Overall, this study illuminates the severity of the acute and chronic illnesses that are commonly seen in tertiary care CICUs and the rate and success of cardiac surgical interventions in this population. Surgical intervention is common, and mortality outcomes are equivalent to medical treatment in the CICU. Due to the critical illness and multitude of comorbidities of these patients, the data show the importance of highly trained specialists comprising a heart team to care for these patients in the preoperative period to improve the likelihood of postsurgical success.

## Funding support and author disclosures

The authors have reported that they have no relationships relevant to the contents of this article to disclose.
